# Medical Waste from COVID-19 Pandemic—A Systematic Review of Management and Environmental Impacts in Australia

**DOI:** 10.3390/ijerph19031381

**Published:** 2022-01-26

**Authors:** Lynda Andeobu, Santoso Wibowo, Srimannarayana Grandhi

**Affiliations:** School of Engineering and Technology, Central Queensland University, Melbourne 3000, Australia; s.wibowo1@cqu.edu.au (S.W.); s.grandhi@cqu.edu.au (S.G.)

**Keywords:** COVID-19, medical waste, waste management, disposal methods, challenges, environmental impacts, Australia

## Abstract

The coronavirus (COVID-19) pandemic has created a global medical emergency. The unforeseen occurrence of a pandemic of this magnitude has resulted in overwhelming levels of medical waste and raises questions about management and disposal practices, and environmental impacts. The amount of medical waste generated from COVID-19 since the outbreak is estimated to be 2.6 million tons/day worldwide. In Australia, heaps of single-use gowns, facemasks/face shields, aprons, gloves, goggles, sanitizers, sharps, and syringes are disposed everyday as a result of the pandemic. Moreover, the establishment of new home/hotel quarantine facilities and isolation/quarantine centres in various Australian states and territories have increased the risks of transmission among people in these facilities and the likelihoods of general waste becoming contaminated with medical waste. This warrants the need to examine management and disposal practices implemented to reduce the transmission and spread of the virus. This study reviews the various management and disposal practices adopted in Australia for dealing with medical waste from the COVID-19 pandemic and their impacts on public health and the environment. To achieve the aims of this study, prior studies from 2019–2021 from various databases are collected and analysed. The study focuses on generation of medical waste from COVID-19, management and disposal methods, current problems/challenges and environmental and public health impacts. Considering the enormous risks involved and the significance of appropriate handling and disposal of medical waste from COVID-19, this study provides insights on short and long term responses towards managing COVID-19 waste in Australia. The study contributes to Australia’s efforts against the transmission and spread of COVID-19 and provides recommendations for the development of workable and sustainable strategies for mitigating similar pandemics in the future.

## 1. Introduction 

The Coronavirus (COVID-19) pandemic has become a threat and a major global challenge [1]. An outbreak of coronavirus disease was discovered in Wuhan City, Hubei Province, China in early December 2019. In late January 2020, the World Health Organization (WHO) declared the outbreak a public health emergency of international concern and subsequently declared it a pandemic in March 2020 [1,2,3]. By May 2020, the virus had spread to more than 188 countries and regions, and as of September 2020, the number of countries infected with coronavirus had risen to 216 [1,4]. Globally, there have been 206,662,675 confirmed cases of COVID-19 and 4,353,058 deaths while 3,984,596,440 COVID-19 vaccine doses have been administered as of 15 August 2021 [1]. As with other countries around the world, Australia is not excluded from the impact of COVID-19 pandemic. The first four cases of COVID-19 in Australia were detected on 25 January 2020. By 15 August 2021, there had been 38,657 confirmed cases of COVID-19 and 953 deaths while 15,012,023 vaccine doses had been administered [5]. Due to the increasing number of infections and mutations of more transmissible variants worldwide, the Australian government has imposed lockdowns, border closures, travel restrictions, and self-isolation orders to reduce the spread of the virus. Other preventive measures such as social distancing, facemasks, and frequent handwashing have been implemented to reduce the transmission of the disease [6]. The WHO and other international health agencies and disease control centres have also issued various guidelines for the prevention of COVID-19 transmission [4,7].

In Australia, the screening of passenger flights from Wuhan to Sydney commenced on 23 January 2020. A few days later, the first four cases of COVID-19 infection in Australia were discovered [6,7,8]. As a result, several border security measures and restrictions on international arrivals entering Australia from China and other countries were put in place. On 3 March 2020, the government issued a notification to citizens and permanent residents of the country travelling for nonessential reasons [7,9]. On 21 March 2020, border restrictions were extended to all other nonpermanent residents and noncitizens [7,10,11]. These border closures and restrictions are ongoing and are anticipated to be gradually lifted in the coming months at an unspecified date [7,10]. With the number of people infected with COVID-19 growing, the first known cases of community transmission were identified on 2 March 2020 [9]. By Friday, 13 March 2020, 156 coronavirus cases had been detected in Australia [7,8]. This escalation in infections prompted the Australian government to commence a 14-day quarantine for both citizens and permanent residents returning to Australia from overseas in publicly funded hotel accommodation under the management and supervision of public health authorities and police [7,8,10]. Currently, some of Australia’s states and territories are in their second, third, and fourth wave since the beginning of the pandemic [10,12].

As Australia responds to the problems and challenges associated with COVID-19 pandemic, management of medical waste generated from COVID-19 has become a major concern [6,7,13]. With the growing number of confirmed cases in Australia, the amount of COVID-19 waste generated in testing sites, hospitals, households, quarantine facilities, and aged/disability care facilities has exponentially increased [6]. In addition to medical waste generated from these sources, the extensive use of personal protective equipment (PPEs) (single-use gowns, facemasks/face shields, aprons, gloves, goggles, sanitizers, sharps, and syringes) has added to the increasing amounts of medical waste from the COVID-19 pandemic [7,14]. Since COVID-19 can survive on surfaces and objects from a few hours to several days, PPE is considered an infectious waste and is treated as medical or hazardous waste [1,2]. Thus, the emergency nature of the pandemic and the response actions that followed has created enormous amounts of infectious COVID-19 waste generation. This warrants the need to examine the management and disposal practices implemented in Australia to effectively manage the virus. Previous studies [6,7,13,15] stated that the transmission and spread of the virus may increase due to inappropriate management and disposal of medical waste generated from COVID-19. Consequently, ensuring that COVID-19 waste are efficiently and safely disposed of has become an important part of the fight against the pandemic.

According to current evidence, transmission of COVID-19 occurs through (a) direct human-to-human contacts, (b) indirect contact with surfaces in the immediate environment, (c) SARS-CoV-2 infective droplets in the atmosphere, and (d) (objects used on infected persons such as thermometer or stethoscope and COVID-19 waste generated from healthcare facilities concerned with diagnosis and treatment [1,16]. Hence, it is important to cautiously manage objects and waste generated from COVID-19 patients. Firstly, the enormous amount of medical waste generated during the pandemic has become a burden [1], and there are high chances of such waste being potentially infected with SARS-CoV-2 virus [2,16]. If not appropriately managed and treated scientifically, there is a high risk of secondary transmission of the virus [2]. The frontline staff such as healthcare workers and waste workers coming into contact with COVID-19 waste could become infected if incorrectly handled [17]. Moreover, uncontrolled dumping in landfills could further elevate the chances of spreading the virus to the general public [18]. Secondly, the COVID-19 pandemic has led to enormous utilization of personal protective equipment (PPEs), particularly face masks and gloves. According to UNEP [2], more than 2.8 billion face masks are expected to be used by 2022. These huge quantities of PPEs are plastics, and their uncontrolled burning would result in release of toxic gases and fumes in the environment. Further, uncontrolled dumping could pollute ecosystems like rivers, beaches, and oceans [2]. Thus, it is necessary to have a well-implemented strategy to deal with COVID-19 waste in order to maintain progress towards achieving various sustainable development goals (SDGs) including Goal-3 (good health and wellbeing), Goal-6 (clean water and sanitation), Goal-8 (decent work and economic growth), Goal-12 (responsible consumption and production), and Goal-13 (climate action) [17,19]. 

This study examines the various management and disposal practices adopted in dealing with medical waste from the COVID-19 pandemic and its impacts on public health and the environment. To achieve the aims of this study, prior studies from 2019–2021 from various databases are collected and analysed. The study focuses on generation of medical waste from COVID-19, management and disposal methods, current problems/challenges, and environmental and public health impacts. In view of the enormous risks involved in managing medical waste from COVID-19 and the significance of proper handling and disposal of these wastes, this study provides insights on short and long term responses towards COVID-19 waste management during the pandemic and also provides alternative management approaches and recommendations for the development of workable and sustainable strategies for mitigating similar pandemics in the future.

While there are prior studies [6,7,10,13,15,20] on the COVID-19 pandemic in Australia, no study has investigated management and disposal practices adopted in handling medical waste from COVID-19 and its impacts on public health and the environment. There are several major issues relating to COVID-19 waste management practices in many countries, including Australia: (a) absence of workable and sustainable medical waste management strategies, (b) lack of adequate knowledge and awareness, (c) absence of strict regulation and legislation, (d) insufficient funding, and (e) implementation [2,3,21]. As a result, the management of medical waste from COVID-19 has become an area of significance to researchers, academics, institutions, and governments. Despite the critical nature of this issue, little attention is attributed to it, particularly in developing and poorer nations [3,22]. Corburn et al. [13], Nzediegwu and Chang [23], and Mihai [24] noted that there is a strong need for further research to be made on COVID-19 waste management practices, the technologies adopted, and the environmental impacts. 

Generally, handling and treatment of infectious medical waste depends on (a) the type of waste, (b) the place of waste generation, and (c) the type of infectious disease [1]. The WHO [18] and UNEP [25] have classified medical waste from COVID-19 as infectious waste. Consequently, tons of infectious medical waste generated every day in Australia from COVID-19 must be handled and managed carefully to prevent the spread of pathogens and to protect public and environmental health, in addition to being separated from other solid waste materials [6,7]. Infectious waste comprises 10–25% of all the waste produced in hospitals and medical centres and cannot be disposed of with the normal domestic waste. According to the WHO [18], infectious waste are defined as infectious waste materials generated as a result of medical care or research containing pathogens that have the potential to transmit infectious diseases. Infectious waste materials can become noninfectious after they have lost infectivity through intermediate treatment, such as incineration, autoclaving, melting, or sterilisation.

According to Capoor and Parida [14], the WHO [18], and Mihai [24], management of COVID-19 infectious waste is a vital and urgent public service and plays a key role in preventing possible secondary impacts on public health and the environment. Hence, special attention should be paid to handling COVID-19 waste during this pandemic [23,24,26]. Hu et al. [27], and Cobourn et al. [28] added that massive amounts of medical waste from COVID-19 are continuously generated and disposed into the natural environment; and inappropriate waste management practices can increase the risk of contamination, particularly in developing countries. In recent years, there has been increasing public awareness on the need to improve medical waste management practices and its segregation from general waste management [1,14,29,30]. Disappointingly, in many countries, efforts to influence governments’ consideration that enormous investments are required to effectively manage medical waste have been unsuccessful [31,32,33,34,35,36]. This study will contribute to creating more public awareness for the adoption of safe and sustainable medical waste management practices directed towards decreasing environmental contamination and reducing the frequency of transmission of coronavirus diseases, particularly in the current COVID-19 pandemic.

A key motivation of this study is to identify the problems/challenges relating to the management of medical waste from COVID-19 and recommend safe and sustainable short and long term solutions for managing medical waste from COVID-19 to significantly reduce transmission and environmental impacts. Accordingly, this study (a) reviews previous research on medical waste from COVID-19 pandemic, (b) identifies problems and challenges that negatively impacts on management of medical waste from COVID-19, (c) provides an overview of management practices in Australia, and (d) identifies areas for future research. This study utilizes outcomes of previous studies, considers Australia’s specific situations, and identifies future research areas to present best practices and recommendations for handling and managing medical waste from the pandemic. 

This research is organized into sections. The first section presents existing literature on medical waste and the COVID-19 pandemic, the problem statement, research aims, and justification for this study. The second section outlines the methodology adopted and the justification for considering a systematic literature review. The third section provides current management practices relating to medical waste generated during the pandemic as well as the environmental and public health impacts. The fourth section presents the results of this study and analysis of the results. The final section provides the findings of this study, limitations associated with the current study, best practices, and recommendations for effectively managing medical waste from COVID-19 and future research opportunities. 

## 2. Research Methods

### Search Strategy and Selection Criteria

This study adopts a systematic literature review (SLR) approach using articles from previous studies on medical waste and infectious waste relating to COVID-19. The purpose of this review is to systematically search high-quality theoretical research materials so as to contextualize the scope of this research and identify research gaps in this study. In conducting the literature search, relevant articles from 2019–2021 were reviewed. Major databases (Science Direct, Springer, Web of Science, Taylor and Francis, Research Gate, Google Scholar) and other major academic publishers were searched. Research papers and the current Guidelines and Recommendation of World Health Organization (WHO), Centres for Disease Control and Prevention (CDCP), United States Occupational Safety and Health Administration (OSHA), and European Union Guidelines for COVID-19 Waste Management and Infection Control Practices were included in this study. Structured keywords such as “medical waste”, “infectious waste”, “COVID-19”, “management and disposal methods”, “problems and challenges”, and “environmental and public health impacts” were used to document research results on topics relevant to this research. The criteria followed in selecting the articles from the databases for this study are as follows:

- Does the paper discuss topics relating to medical waste from COVID-19?
- Does the paper discuss generation and treatment of medical waste relating to COVID-19?
- Does the paper discuss topics relating to management and disposal of medical waste from COVID-19?
- Does the paper discuss problems and challenges of medical waste from COVID-19?
- Does the paper discuss topics relating to COVID-19 and public health?
- Does the paper discuss topics relating to environmental impacts of COVID-19 waste?

**Figure 1:**
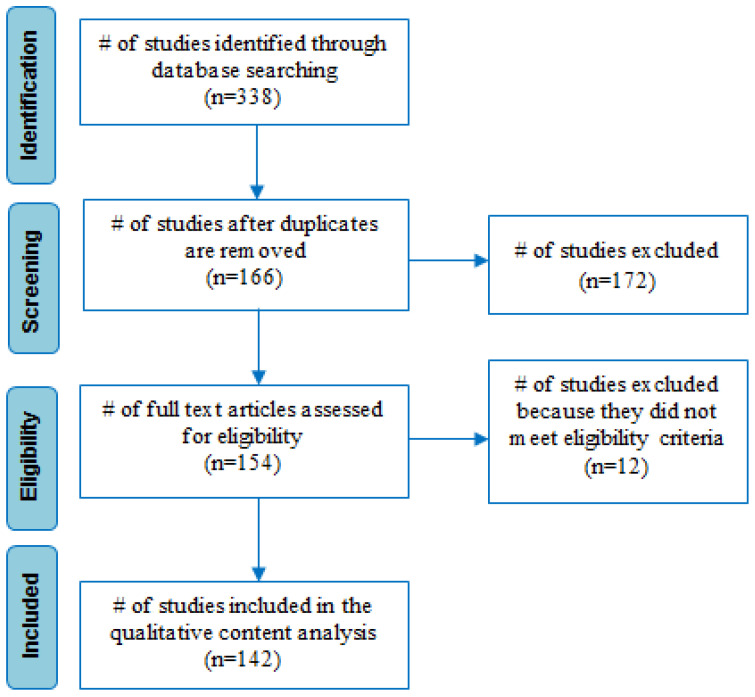
PRISMA flow chart indicating the results of searches.

Initially, 338 articles were obtained, although some were excluded because they analysed explicit topics that were beyond the scope of this research. Finally, 142 articles were reviewed for this study, taking into consideration aspects such as the language in English, year of publication, location of study, scope/type of study, and methodology. From a large number of the articles selected, this study collects, analyses, and presents information relevant to the management, disposal, challenges, and environmental and public health impacts of waste from COVID-19 that meet the aims of this study. Figure 1 shows the process for selecting and rejecting papers based upon these eligibility criteria.

## 3. Overview of Medical Waste 

Across the world, the management and disposal of medical waste is of growing concern. This is due to the hazardous nature of medical waste and the potential threat to humans and the environment [1,37,38]. While medical care is critical to our wellbeing and saves lives, the waste generated from medical practice in hospitals, clinics, age care facilities, health care teaching institutes, research institutions, blood banks, laboratories, and veterinary institutes can be harmful due to their high potential to transmit diseases and contaminate the environment [30,37,39]. According to Wilder-Smith and Freedman [40], Malekahmadi and Yunesian [32], and USEPA [41], medical waste from human activities has been identified as the third largest known source of dioxin emission and contributes 10% of total mercury emissions to the environment. In the past few years, the generation of medical waste has significantly increased. This has been intensified with the emergence of COVID-19 pandemic [42]. As a result, management and disposal of medical of waste continues to be a major challenge in many parts of the world, including Australia. According to Peng et al. [42] and Sutha-Irin [43], some of the impediments of managing medical waste are (a) lack of awareness among healthcare professionals and the general public regarding inappropriate handling of medical waste, (b) the absence of a national policy and regulatory framework, and (c) financial constraints. All of these factors increase the risk of environmental and public health vulnerabilities [18]. With the COVID-19 pandemic escalating and its impacts on public health and the economy intensifying each day, governments are advised to treat medical waste from COVID-19 with caution to reduce the transmission and spread of the virus [3,10,14,42].

### 3.1. Definition and Classification of Medical Waste

Medical waste is generated from the diagnosis, treatment, or immunization of humans and animals. According to the WHO [1] and EPA South Australia [44], medical waste is defined as waste consisting of any matter that is discarded in the course of medical, dental, veterinary practice, or research and that poses a significant risk to the health of a person who comes into contact with it. Medical waste generated from healthcare activities includes needles and syringes, soiled dressings, body parts, diagnostic samples, blood, chemicals, pharmaceuticals, medical devices, and radioactive materials [45].

Medical waste is classified into two categories, namely, hazardous and nonhazardous waste [1,34,35,46,47,48]. The classification of medical waste indicates that (a) of the total amount of waste generated from healthcare activities, about 80% is general waste; (b) the remaining 20% is considered hazardous material that may be infectious, toxic, or radioactive; (c) approximately 16 million injections are administered yearly worldwide and waste generated are not appropriately disposed; and (d) medical waste contains harmful micro-organisms that can infect hospital patients, healthcare workers, and the general public [1,33]. Hazardous waste is categorized on the basis of the risk of infection and injury during its management and disposal process. These categories include sharps (blades and contaminated needles), infectious waste (blood, body fluids, and dressings), and pathological waste (microbiological cultures, blood samples, and anatomical body parts) [18,49]. Hazardous (infectious) waste often contain pathogens such as bacteria, fungi, viruses, and parasites in adequate concentration, and can cause disease in vulnerable hosts [50,51].

Prepandemic, policies on medical waste targeted only the medical community; thus, public awareness and consciousness on medical waste are insufficient [14]. The onset of the current COVID-19 pandemic has created the need for public awareness and the importance of its separation from general waste, which needs to be addressed to control coronavirus transmission [6]. Table 1 provides medical waste categories and their examples as defined by the WHO. 

Generally, some categories of medical waste, including those from COVID-19 patients, are classified as infectious waste and are highly transmissible [16,52]. Waste generated from hospitals, laboratories, testing centres, households quarantine facilities, graveyards, and crematoriums, in which a person infected with COVID-19 virus has been tested, isolated, or treated are categorized as COVID-19 waste [53,54]. In the past two years, the risk categorization of COVID-19 waste has been changing due to various concerns as the pandemic progressed. While there are other categories of medical waste, infectious waste, including those from COVID-19, are particularly harmful because they contain pathogens that possess the risk of infection to humans with long-term effects [27]. Due to the ever-increasing number of COVID-19 infections worldwide, the dangers of COVID-19 transmission through (a) direct and indirect human-to-human contacts, (b) SARS-CoV-2 infective droplets in the atmosphere, and (c) COVID-19 waste from healthcare facilities concerned with diagnosis, research, or treatment and waste generated by COVID-19 infected or suspected persons isolated/treated in households or other facilities have become apparent [1,25,27,55]. 

Prior studies of the WHO [18], Graff et al. [56], Qiao [57], Scheinberg et al. [58], Chen et al. [59], and Salvaraji et al. [60] have focused on assessing the risks of transmission associated with medical waste from COVID-19 pandemic. The inappropriate treatment of COVID-19 waste poses serious risks of transmission to the general public, particularly waste workers, healthcare workers, and patients, through exposure to infectious pathogens [56,58,59]. In addition, poor management of COVID-19 waste emits harmful contaminants into the environment [61]. Hence, environmental risks and occupational risks assessment due to COVID-19 waste has become an important part of handling and reducing the risk of transmission [57,59,62].

To ensure the provision of treatment and medical care to the rising number of COVID-19 patients, healthcare infrastructure around the world has been largely expanded [19]. People in various parts of the world are now using face masks and gloves to protect themselves from contracting COVID-19 virus [19,54]. These swift changes over a short period of time have resulted in a sudden increase in the quantity of COVID-19 waste meant for disposal [25]. For example, some countries have reported up to a 10–20 times upsurge in waste generated during the pandemic when compared with those generatedbefore the pandemic [45,55,63]. Clearly, the existing waste management systems worldwide, including those in Australia, are under pressure and are not designed to handle such a surge of waste generated; hence, the safe handling and disposal of COVID-19 waste is viewed as one of the biggest risks of the current pandemic [18,19,63].

Prior to the current pandemic, access to waste collection was not available to more than two billion people worldwide, while approximately three billion lacked controlled waste disposal facilities; this reflects the state of waste management systems in many parts of the world, particularly in developing countries [25,54]. With the increase in COVID-19 waste generated, cases of open burning and uncontrolled disposal of waste cannot be overlooked [1]. This has, in turn, led to higher risk of pollution, littering, and environmental degradation, including increased possibilities of secondary transmission of the virus from COVID-19 waste [18,64,65].

Waste management and disposal is an essential service, which is continuously required across cites, even in areas categorized as hotspots for COVID-19 [1,54]. Safety of waste handlers should be given priority; the role of waste handlers has been classified under essential services by various countries because timely and proper collection, treatment, and disposal of waste are essential during the pandemic [25,58]. In developing countries, where waste handlers are not equipped with needed PPEs, the extent of risk is often higher [58]. Moreover, due to the nature of waste generated from COVID-19 patients in home isolation/quarantine, which is often mixed with other municipal waste, members of a household are at risk of contracting COVID-19 infection from incorrectly dumped infectious waste such as used face masks, gloves, and other infectious municipal waste [56].

Further, research on the risk of COVID-19 transmission has revealed that the life span of SARS-CoV-2 droplets containing the virus survive for almost 3–7 days on surfaces and wastes from plastics, stainless steel, copper, and cardboard. This has resulted in concerns regarding contaminated face masks and gloves that are thrown away as general waste [56]. Thus, the transmission of highly contagious pathogens such as the COVID-19 virus has created enormous pressure on medical waste management, treatment, and disposal due to the volume of waste generated daily [18,65]. This has prompted many countries, including Australia, to adopt safety measures to fight the disease through efficient management and disposal [25]. The WHO and other international organizations have set guidelines for management of medical waste, including COVID-19 waste, as discussed in Section 4. These guidelines help to manage the highly contagious COVID-19 waste emanating from the current pandemic [22].

The scientific community and governments across the world had little knowledge about the nature and extent of the pandemic and its associated impacts [18]. To ensure that waste management systems are robust and resilient during the pandemic, comprehensive information about COVID-19 waste generation must be ascertained with strict compliance on the recommended practices and guidelines for management and disposal [53,63]. 

Consequently, risks from COVID-19 infectious waste have been highly rated and categorized as hazardous, requiring careful treatment and disposal during the pandemic. These risks are particularly critical since vaccination protocols are still in the developmental phase and there are many countries across the world with low vaccination rates [51,66]. An overview of medical waste policy and the regulatory framework in Australia is presented in the next section.

### 3.2. Overview of Australia’s Medical Waste Policy and Regulatory Framework

There are 168 national laws and regulations that address waste management worldwide [50]. Of the 168 laws and regulations, only 57 deal with medical waste streams, while the remaining 111 address other waste streams [31]. In Australia, there is no national law or regulation on medical wastes. At the national level, the National Waste Policy specifies the general policies and strategies on waste, one of which is to collaborate with relevant jurisdictions to create a national definition and classification system for wastes, including hazardous and nonhazardous medical wastes, that aligns with international conventions. At state and territory levels, each state/territory has “put in place its own policies and regulations that deal with the management of waste (including medical waste), which also aligns with international conventions” [67,68]. For example, the Clinical and Related Waste Management Policy of 2016 in Western Australia addresses both the hazardous and nonhazardous waste streams of medical waste [69]. In South Australia, the Environment Protection (waste management) Policy 1994 addresses all categories of waste (excluding of radioactive waste) and is regulated under the Radiation Protection and Control Act 1982 (EPA South Australia, 2003). To harmonize and to support safe and cost-effective medical waste management practices in transportation, storage, treatment, and disposal, the healthcare industry in collaboration with the waste management industry have developed the Code of Practice for the management of medical wastes [67,70]. Guidelines and procedures for the management of COVID-19 waste recommended by the WHO and other international organizations for treating and disposing of potentially infectious COVID-19 waste are discussed in the next section.

## 4. Current Medical Waste Management Guidelines of Regulatory Agencies

Globally, the waste management and recycling sector has been greatly impacted by the current COVID-19 pandemic due to large-scale quarantine, patient care, diagnosis, treatment, and isolation [50,54,64]. While the major source of medical waste from COVID-19 are from healthcare establishments such as hospitals and laboratories, other sources could be home quarantine facilities, temporary establishments, sample collection and testing centres, graveyards, and crematoriums [17,54]. Due to the enormous medical waste produced during the current pandemic, the WHO and other international organizations including the European Centre for Diseases Prevention and Control (ECDC), United States Occupational Safety and Health Administration (OSHA), Basel Convention Regional Centre for Asia and the Pacific, and Central Pollution Control Board (CPCB) have provided guidelines and practices for managing medical waste including COVID-19 waste to prevent and/or reduce the risk of infection.

### 4.1. World Health Organisation (WHO)

The provision of safe water, sanitation, waste management, and hygienic conditions is vital for preventing and protecting human health during outbreaks of all infectious diseases including the current coronavirus disease (COVID-19). Regularly applying water, sanitation, hygiene (WASH), and waste management practices in communities, healthcare facilities, homes, schools, marketplaces, and other public places will help prevent transmission of infectious pathogens including SARS-CoV-2. WHO stated that application of WASH and appropriate waste management practices are critical to preventing and/or reducing the spread of COVID-19, and issued guidelines for their implementation [61].

The recommendations of the WHO for WASH and waste management in healthcare settings are particularly essential for providing adequate care for patients and protecting healthcare workers, waste handlers, caregivers, and patients from the risks of infection. According to the WHO [71], while the WASH recommendations will not prevent COVID-19 transmission, following standard WASH related actions such as (a) engaging in frequent hand hygiene using appropriate techniques, (b) implementing regular environmental cleaning and disinfection practices, (c) managing excreta (faeces and urine) safely, (d) safely managing medical waste produced from COVID-19 cases, and (e) safely managing dead bodies are particularly critical.

The WHO [71] states that additional treatment or disinfection or prescribed waste management procedures are not required specifically for COVID-19 waste since waste arising from healthcare facilities with or without COVID-19 patients are not different. The WHO emphasized appropriate segregation of waste since general and noninfectious waste are a major component of waste in healthcare facilities and can be disposed of as general municipal waste. They also recommend on-site treatment of infectious medical waste generated in the course of treatment of patients, preferably through high temperatures, autoclaving, and dual-chamber incineration.

In addition, the WHO advocated the need for expanding waste treatment infrastructure using alternate and more efficient technologies due to the sudden surge in the quantity of waste generated, especially the discarded PPEs. Safe burial of healthcare waste may also be practiced, as an interim solution, until more sustainable options are implemented. However, manual disinfection is not a recommended option for treating waste since it is unreliable and inefficient [61].

### 4.2. European Centre for Diseases Prevention and Control (ECDC)

ECDC is aimed at strengthening defence and control against infectious diseases including COVID-19 disease. Excerpts of guidelines issued by the European Union based on the advice of the European Centre for Diseases Prevention and Control (ECDC) with respect to medical waste including COVID-19 waste management are as follows:

A waste bag should be available in the patient’s room that is quarantining and self-isolating at home. Items such as paper tissues, gloves, masks, and other wastes discarded by the patient must be promptly placed in the bag. Another waste bag should be for the caregiver for discarding his/her used face masks and gloves. These bags shall be placed in a separate garbage bag. 

Waste arising from healthcare facilities, laboratories, and other activities related with COVID-19 patients should be managed according to the provisions of EU law on medical waste and relevant national regulations related to infectious waste as well as instructions delivered by ECDC and national health authorities [72]. 

### 4.3. Occupational Safety and Health Administration (OSHA)

The Occupational Safety and Health Administration of the United States have also issued advice with respect to the management of medical waste including COVID-19 waste [73]. Excerpts of guidelines issued by the Occupational Safety and Health Administration are as follows:

According to OSHA, infectious medical waste related to COVID-19 should be managed as regulated medical waste, for which the Centres for Disease Control and Prevention (CDCs) Guidelines for Environmental Infection Control in Health-Care Facilities are to be referred. 

OSHA recommends placing and containing the medical waste in a leak-proof and sturdy biohazard bag for disposal. For containment of sharps, syringes, and needles, puncture-proof containers are recommended to be used. OSHA also advocates following standard engineering and administrative controls, safe working practices, and use of PPEs to prevent exposure of workers to any type of infectious waste as well as any contaminants in the waste being managed. Such controls would safeguard workers from injury due to sharps or other items that may cause injury or expose the workers to infections. Contaminated or potentially infectious general waste (municipal waste) should be managed similarly to that of noncontaminated municipal waste [74].

### 4.4. Basel Convention Regional Centre for Asia and the Pacific

The Basel Convention Regional Centre for Asia and the Pacific published the detailed guidelines titled Handbook of Emergency Disposal and Management of Medical Waste for the management of medical waste during the COVID-19 pandemic and the centre recommends the following:

- Waste should be packed and sealed in double-layer bags and properly labelled.
- Collection of waste generated from clinics and ward rooms for treatment and diagnosis of COVID-19 patients shall be as per the assigned classification of medical waste.
- Waste generated in households by COVID-19 patients such as used masks, gloves, tissues, and other wastes shall be managed as medical waste, collected in sealed bags, and disposed through incineration [65,71].
- For emergency disposal during the COVID-19 pandemic, the waste should be disposed based on category and through decentralization. Waste not meant for emergency disposal should be disposed through centralized medical waste disposal facilities. Waste PPEs generated from COVID-19-related activities shall be treated and managed as medical waste. They shall be segregated as per the prescribed standards and collected and disposed within 24 h [61].

### 4.5. Central Pollution Control Board (CPCB)

The Central Pollution Control Board (CPCB) published the Guidelines for Handling, Treatment and Disposal of Waste Generated during Treatment/Diagnosis/Quarantine of COVID-19 Patients. The guidelines detail responsibilities and duties of various stakeholders involved in the management of medical waste, from generators to regulatory bodies. Excerpts of guidelines issued include the following:

- General waste generated in healthcare establishments should not be mixed with biomedical waste. It should be collected and managed separately in accordance with the provisions of the Solid Waste Management Rules 2016.
- For quarantine camps, home quarantine, and home-care facilities where COVID-19 patients are isolated for treatment, only used masks, gloves, swabs, tissues, syringes, etc. shall be handled as biomedical waste and stored in yellow-coloured bags.
- Isolation wards and temporary medical establishments should keep separate colour-coded, foot-operated bins for segregation of biomedical waste, including COVID-19 waste, as per the provisions laid down in the Bio-medical Waste Management Rules 2016.
- Dedicated bins labelled as ‘COVID-19 Waste’ should be used. Double-layer bags should be for medical waste collection [75].

The guidelines and recommendations of the WHO and other international organizations have been widely implemented in various countries, including Australia. While certain procedures and techniques for collection, treatment, and disposal of COVID-19 waste vary from country to country, the basic underlying principle of their management are the same as most countries’ national legislations on medical waste. They are based on the following: (a) safe management of waste from medical care activities—A WHO Publication; (b) Technical Guidelines on the Environmentally Sound Management of Biomedical and Healthcare Waste, adopted in 2002 under the Basel Convention; and (c) Water, sanitation, hygiene (WASH), and waste management for SARS-CoV-2, the virus that causes COVID-19.

In Australia, the National Biohazard Waste Industry (BWI) committee, a division of the Waste Management and Resource Recovery Association of Australia (WMRR), is the peak body for the management of medical and related waste in Australia. In the wake of the declaration of COVID-19 as a pandemic by the World Health Organization (WHO), the committee developed additional guidelines based on WHO recommendations to assist in providing direction to hospitals, aged care facilities, and healthcare providers in managing COVID-19-affected materials, and to support those managing medical waste, both within and outside of these facilities [67]. The next section presents COVID-19 waste management and disposal practices in Australia.

## 5. COVID-19 Waste Management and Disposal Practices in Australia

**Figure 2:**
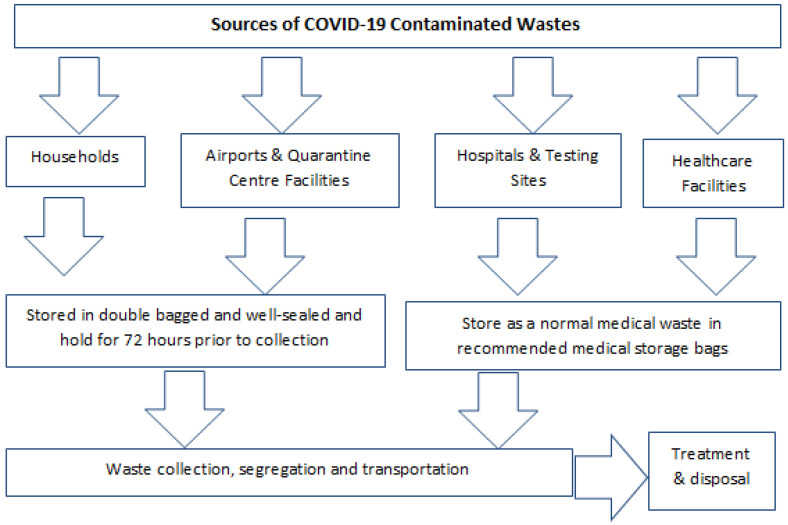
Sources of contaminated waste from COVID-19 pandemic and paths for safe management and disposal. Image developed for this study. Data adapted from the WHO [1] and Australian Government Department of Health [76].

As COVID-19 virus continues to spread globally, international organizations such as the WHO and UNEP have emphasised the need to manage and dispose medical waste from COVID-19 with care to minimize the risk of unending transmission of the virus [1,66]. With Australia’s stay at home orders and strict lockdowns in some states and territories, the nation is facing increases in household and medical waste. This has created challenges and put enormous pressure on waste management and disposal agencies [7,10]. Ensuring that waste management services in Australia’s states and territories are keeping up with industry and local standards in terms of safety, hygiene, and efficiency is therefore critical [7,15,75,76]. The management and disposal of coronavirus-related wastes commences with appropriate estimation of the amounts generated. Figure 2 shows the sources of contaminated waste from COVID-19 pandemic and safe management and disposal. 

### 5.1. Generation 

In other to adequately manage medical waste, relevant information on healthcare waste generation is necessary to support the formulation of waste management strategies [29,31,77]. The amounts of medical waste generated are used to estimate the required capacities for (a) containers, (b) storage areas, and (c) transportation and treatment technologies. It also assists in establishing a baseline on rates of generation in different facilities, procurement specifications, planning, budgeting, and revenue requirements for treatment, recycling, optimization of waste management systems, and environmental impact assessments [77,78,79].

In Australia, a number of factors are responsible for the variations in COVID-19 waste generation and these include (a) the type of facility, (b) the size of the facility, (c) the number of people receiving treatment, and (d) the type of treatments provided at the various facilities [42,77]. Prior to the pandemic, in 2019, Australia produced about 67 million tons of medical waste [6,15]. Due to the pandemic, the amount of medical waste generated globally has significantly increased [16,23,42]. Currently, in Australia, on average, medical waste generated from COVID-19 fills one 240-litre wheelie bin per week. States and territories with higher numbers of active cases of coronavirus are now filling as many as twelve of 240-litre bins per day, with an approximate of 84 bins per week [6,10]. Thus, effective management of medical waste from COVID-19 and other health-care waste requires appropriate methods of collection, segregation, storage, transportation, treatment, and disposal [25,42,45,77], and these are discussed below.

### 5.2. Collection

In Australia, medical waste generated from other patient care and COVID-19 are collected by local waste collection agencies and guided by the state and local policies and procedures [76]. All medical waste generated from confirmed COVID-19 infections are classified as infectious waste and should be clearly marked in lined containers and sharp-safe boxes for collection [69,80,81,82]. The infectious COVID-19 contaminated waste generated should be adequately disinfected and then separated and packed in their respective recommended waste disposal bags [18,19]. To ensure adequate protection, double-layered leakproof bags are to be used for the collection of waste generated from COVID-19 patients [69,80]. These COVID-19 waste containers are not to be kept in public areas to prevent the possibilities of contamination when others use the same containers [18,80]. 

When waste collectors are collecting COVID-19 waste from hospitals, testing sites, healthcare facilities, laboratories, and infected patients under quarantine, they are to be provided with appropriate PPEs [45]. Based on medical waste management policies and regulations, red bags are to be specifically used for the collection of PPEs such as face masks, face-shield, goggles, aprons, plastic gowns, and gloves [1]. Nonchlorinated yellow plastic bags are to be used for collection of bedding contaminated with body fluids or blood. Since waste from COVID-19 are not only generated from hospitals, but also from other sources such as testing sites, airports, quarantine centres, and households, COVID-19-contaminated waste produced from these sources are to be collected separately in yellow-coloured bags for local waste collection agencies. After collection, adequate hand hygiene should be performed to avoid virus transmission [1,80].

### 5.3. Segregation

One of the most crucial phases of medical waste management is the segregation phase [49,50,82]. In many developed countries, including Australia, the rules and implementation of medical waste segregation are widely practiced [51]. This scenario is different in developing countries. For example, prior studies [31,36,83] on Asian developing countries reported that there is lack of appropriate compliance in segregation and the implementation of standards, creating variations from country to country. Khan et al. [31] asserted that absence of proper medical waste segregation could increase the volume of infectious waste, which has the potential to convert general waste into infectious waste. 

Segregation is useful since it prevents the contamination of nonhazardous waste by hazardous waste, thereby making the whole waste stream hazardous. Moreover, appropriate segregation will reduce the toxicity and volume of the waste streams and make it easier to transport. Waste is segregated depending on the quantity, composition, and disposal method of the waste stream [66,71].

Many countries have national legislation that prescribes the waste segregation categories to be used and a system of colour coding for waste containers [18,66]. According to the standard rules and regulations in Australia, medical waste are to be contained in colour-coded and labelled bags or containers [84]. Medical waste from COVID-19 are categorized by the colour “yellow”, incinerated, and cannot be disposed of into the general waste or recycling waste streams [80,84]. Colour coding makes it possible for hospital staff handling waste to place waste items into the right containers with ease and to maintain segregation of the wastes during transport, storage, treatment, and disposal. Colour coding also helps to provide a visual indication of the potential risk posed by the waste in a particular container [2,4,45]. 

When disposing nonsharp medical waste from COVID-19 such as contaminated PPEs, they should be placed in a yellow biohazard bag, which complies with Australian Standards [5,70]. These yellow biohazard bags should be tied off at the point of segregation and disposed of into a yellow 2-wheeled mobile garbage bin (MGB). These bins should be stored according to the prescribed standards. Medical waste from COVID-19 generated at the airports can be disposed of in quarantine bins and should refer to local policy [81]. Medical sharps from COVID-19 are not to be placed directly into MGBs. Instead, sharps must be disposed of into designated sharps containers that meet Australian Standards. These sharps containers must not be overfilled and should be sealed when it reaches the designated fill line [5]. 

### 5.4. Storage and Transportation

Transportation is an important aspect of managing medical waste from COVID-19. Before transportation, all waste contaminated with COVID-19 must be sealed in bags. The bags must be properly labelled/barcoded before transportation [1,80]. The waste materials from the collection site should be transported to the storage area in a separate trolley labelled with COVID-19. The trolleys used for transportation of COVID-19 waste are to be disinfected with 1% sodium hypochlorite after delivery [1]. For safe transportation, an appropriate transfer route; a dedicated trained driver; and a designated vehicle are to be used [69,80]. In addition, on-site transport of COVID-19 waste should take place during off-peak times whenever possible. Established routes should be used to prevent staff and patients from being exposed and to minimize the passage of the loaded trolley through patient care and other clean areas [80]. Specific and regular transportation routes as well as collection times should be fixed. Most of the states and territories have introduced a manifest system/tracking system when transporting COVID-19 waste off-site. The following information are often documented: (a) waste type, (b) waste sources, (c) pick-up date, (d) destination, (e) driver’s name, (f) number of containers, (g) receipt of waste from responsible person at the pick-up area [18,67].

**Figure 3:**
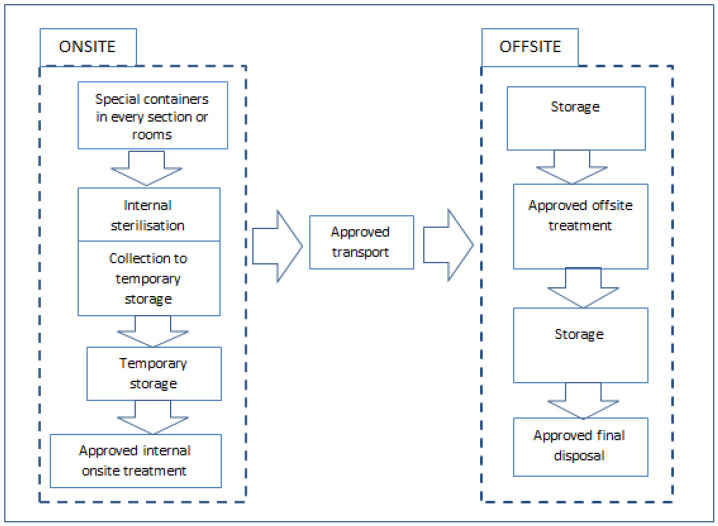
A flow chart of COVID-19 waste management. Developed for this study. Data adapted from the UNEP [66] and WHO [61].

COVID-19 waste and general waste should not be collected at the same time. All workers coming into contact with COVID-19 must be adequately protected with required PPEs [18]. A designated storage area located away from patients and public access should be established. It should also be well secured, ventilated, and inaccessible to pests. A separate waste storage bin labelled with COVID-19 should be used to store and keep COVID-19 waste in a special temporary storage room to avoid mixing with other types of wastes; this will help the waste treatment staff to easily identify the waste and treat it accordingly. COVID-19 waste should be disinfected (0.5–1% chlorine solution) immediately after waste delivery and not be stored for more than 24 h after collection [69]. Figure 3 shows a flow chart of COVID-19 waste management [1,25].

### 5.5. Treatment and Disposal Methods

Medical waste, particularly those contaminated with COVID-19, must be treated by strictly following approved guidelines and regulations [1,2] (The objective of COVID-19 waste treatment is to make the waste as safe as possible for hospital staff and the general public and to minimize environmental impact. Generally, any method used for the treatment of COVID-19 waste or other infectious medical waste in Australia should (a) render the waste noninfectious, (b) render the waste unrecognizable, (c) significantly reduce the volume of the waste, (d) not result in unacceptable levels of hazardous or toxic by-products, and (e) be environmentally acceptable [69,80,85]. A number of methods are used for the treatment and disposal of COVID-19 waste and other infectious medical waste. The treatment and disposal methods adopted depends on a number of factors such as the source of the waste, the various components and hazards linked with the waste stream, and the capabilities and limitations of the technology adopted to treat the waste. The treatment methods used should be armed with automated process controls, including continuous monitoring, recording, and shutdown mechanisms [1,66,69]. 

There is no single medical waste treatment technology capable of treating all categories of medical waste. However, waste management agencies should evaluate treatment alternatives based on their safety, effectiveness, environmental impact, costs and compliance with required standards [18,66]. Thus, it is the duty of the waste generator to have approved processes in place and ensure that waste segregation is compatible or suitable for the proposed treatment or disposal technology adopted [18,19,69]. Some of the treatment and disposal options used to manage medical waste including COVID-19 waste are as follows: (a) approved landfill, (b) autoclave (steam sterilization) and shredding, (c) chemical disinfection, (d) grinding/shredding (with sodium hypochlorite), (e) grinding/shredding (with hydrogen peroxide and lime), (f) microwave disinfection and shredding, and (g) approved incineration [66,69,85]. Table 2 provides a summary of the various treatment and disposal methods for medical waste, including COVID-19 waste.

### 5.6. Emerging Technologies for the Treatment and Disposal of Medical Waste

The growing mountain of medical waste (including COVID-19 waste) is creating considerable public health and environmental challenges worldwide (see Section 6). The situation is worrying in less-developed countries due to inappropriate treatment and disposal methods, inadequate physical resources, and lack of research on medical waste management [1]. Although treatment and disposal options shown in Table 2 have been effective in managing medical waste (including COVID-19 waste), new and improved technologies that transforms medical waste into solid waste have been recently introduced [66]. Some of the more recent treatment technologies include (a) irradiation (e.g., UV, Cobalt 60, electron beam), (b) thermal (dry heat involving quartz infrared or plasma pyrolysis), and (c) other inactivation mechanisms (including electrothermal deactivation and plasma gasification) [19,61]. 

## 6. Challenges of Managing COVID-19 Waste

The current COVID-19 pandemic has presented new challenges in almost every aspect of our daily lives and activities [4,86,87]. The pandemic has had widespread effects on countries globally and created a huge burden on healthcare systems [86,88]. Apart from the health-related effects of COVID-19, the pandemic has impacted on the economies of many nations, including Australia. Due to the current COVID-19 pandemic, Australia has seen a radical increase in the use of facial masks and other PPEs, plastic disposables, sanitising liquids, and disinfectants [7]. With the current upsurge in the number of confirmed cases, particularly in Sydney and Melbourne, the amount of COVID-19 waste generated, including infected face masks, face shields, gloves, gowns, and other noninfected waste have increased substantially. This has swiftly changed the dynamics of medical waste management in the states and territories [7] Accordingly, it is critical that federal and state governments ensure that waste management agencies are keeping up with industry and local standards and procedures to reduce the transmission and spread of coronavirus disease [10,15]. 

According to ADB [4], a COVID-19 patient can generate on average around 3.4 kg of medical waste/day, and this has gradually increased in quantity as the pandemic has progressed. Indeed, a Sydney local council reported a 35% increase in medical waste due to the pandemic [10,15]. Other countries are facing similar challenges, in fact, during this pandemic, medical waste generated in the Hubei Province in China increased from 40 tons/day to 240 tons/day (an increase of 600%) [4,89,90]. In the USA, UK, and Japan, governments are currently facing challenges in retaining waste management staff, medical staff, maintaining a COVID-19-safe environment for workers, handling COVID-19 waste generated by quarantine patients at home, and creating space for additional medical waste generated by the pandemic [14]. Currently, PPEs are utilized by the general public including households, care centres, testing sites, staff in public and private offices, airport staffs, and railway staff. These new users are either unaware or untrained on the potential risk of inappropriately disposing of PPEs [77]. 

Apart from the challenges in managing medical waste from coronavirus (PPEs and other COVID-19-related waste), the pandemic has also caused extensive job losses and threatened the wellbeing of millions of people as businesses have shut down to control the transmission and spread of the virus. Around the world including Australia, flights have been cancelled and transport systems have been impacted [7]. In addition, there are a number of environmental problems that have arisen due to the large amounts of COVID-19 waste generated that need to be addressed [6]. 

Although Australia has one of the most affordable, comprehensive, and accessible healthcare systems in the world, the nation is facing challenges in the management and safe disposal of COVID-19 waste due to the unforeseen nature of the current pandemic [6,7]. With contaminated objects rapidly becoming hosts for the transmission and spread of coronavirus, healthcare providers must ensure that medical waste wastes generated from COVID-19 are managed and treated with caution and in compliance with strict federal and state-based guidelines and regulatory requirements. Maintaining and enhancing Australia’s standard of care during this pandemic requires safe and efficient waste management solutions that can cope with the enormous COVID-19 waste generated [6,15]. Thus, as the management of medical waste is closely regulated by state and federal governments, healthcare providers, quarantine facilities, testing sites, and aged care facilities must adopt sustainable, compliant, safe, and reliable practices to deal with COVID-19 waste challenges. Effective and safe COVID-19 waste management will not only help reduce the transmission and spread of the virus, but will also support regulatory and compliance requirements, protect public health, meet corporate social responsibility (CSR) requirements, improve operational efficiency, and keep costs low [7,15]. 

## 7. Environmental and Public Health Impacts

The COVID-19 pandemic has impacted on the environment and public health both directly and indirectly, and assessing these impacts is an enormous task [26,86]. The environmental impacts resulting from the current pandemic include changes in (a) traffic and mobility trends, (b) air pollution, (c) noise level and pollution, and (d) waste generation [6,91]. Worldwide, lockdowns and restrictions have also led to reductions in global emissions and drastically changed patterns of energy demand [59,86]. However, regardless of the adverse socioeconomic impact, COVID-19 is perceived as a blessing in disguise in some instances because it effectively reduced the level of environmental pollution [92]. For example, air travel fell to the lowest in 75 years by about 96%, resulting in a reduction of environmental pollution by up to 30% with a positive impact on environmental quality [86,93,94]. As with the transportation sector, manufacturing and industrial plants, which significantly contribute to environmental pollution, saw a decline in activities and reduction in emissions due to restricted economic activities [63,86]. 

In Australia, the mobility and traffic trends for workplaces, retail and recreation centres, public transport, hubs, supermarkets, pharmacies, and parks plummeted significantly throughout the first and second waves of COVID-19 [6]. These reductions in mobility and traffic trends were as a result of enforced lockdowns leading to mainly working from homes; staying within 5 km from homes; and closing of pubs, shops, retails, and recreation venues. Moreover, during the lockdowns, social distancing restrictions and peak-hour bottlenecks in most congested areas of Australia (e.g., Melbourne and Sydney) vanished [6,7]. Prior studies [17,65,95,96] show that daily global CO_2_ emissions decreased by 17% in April 2020 compared with the previous year, although pollution levels have started rising again as economies recover. For example, during the lockdowns in Victoria, imposing stringent social distancing measures and curfew policies required people to leave their homes for shopping within specific times, returned students to remote learning, and obligated many people to work from home [6]. When compared with the first lockdown in Australia, harder restrictions during the second lockdown led to a reduction in CO_2_ emissions; some public transport services; traffic levels; and the closure of many retail stores, restaurants, and factories in several areas. These changes led to a reduction in environmental noise levels and pollution in affected states and territories [7].

Further, during the lockdowns, due to social distancing and curfew policies, it has been observed that greenhouse gas emissions reduce to record levels [6,97]. According to UNCTAD [98], the global pandemic and the lockdowns that followed led to a 5% decrease in greenhouse gas emissions in the first half of 2020 when compared with the same period in 2019. Liu et al. [93] reported that global CO_2_ emissions reduced by approximately 8.8% for the same period. In China, considerable reductions in nitrogen dioxide levels were observed in major cities due to shutdowns of some industrial facilities and power plants, strong social distancing measures, and curfews [99]. Similarly, in Europe, it has been reported that air pollution and noise levels have significantly reduced [100,101].

Since the start of the pandemic, efforts to reduce single-use plastics seem to have overturned. This is largely due to the increased use of plastic packaging materials, a surge in online shopping and deliveries, and a shift to buying take-away packaged food instead of eating in restaurants [6,102,103,104]. The high demand for plastic products observed during the pandemic led to the suspension of policies on the production of plastic packing materials [17,54]. The suspension is mainly due to (a) increased demand for PPEs in the healthcare system and communities, (b) the recommended or mandatory policies on face masks around the world, and (c) the need for plastic packaging materials due to increased online shopping and home delivery services [54,96]. Thus, much of the disposable plastic waste generated from PPEs during the pandemic are recyclable; however, due to fear of virus transmission, PPEs from medical staff and COVID-19 patients in hospitals and at home cannot be recycled [54,98,105]. 

In addition, the COVID-19 pandemic has triggered huge pressure on landfills in many countries, particularly in developing countries, where solid waste materials and hazardous waste are dumped in poorly managed and open landfills, leading to threats to public health and the environment [96]. The WWF [95] stated that the majority of the world’s discarded waste from COVID-19, particularly PPEs, ends up in landfills, rivers, and oceans, compounding the existing plastics threat to the planet’s ecosystems. According to a study conducted by the WWF [95], if 1% of face masks generated during the pandemic are inappropriately disposed of, about 10 million face masks will end up polluting the environment each month. 

Another environmental concern due to the pandemic is the shedding of coronavirus in stool, which could lead to contamination of community drinking, surface and ground waters if sewage lines are not properly constructed or fail to operate properly [27,106,107]. Sewage overflow during heavy rainfall can cause human exposure from sewage water. Moreover, there are concerns of the long-term effect on COVID 19 viral RNA shedding in faecal matter in patients, which could have implications for public health and the environment [14,27,52]. 

Thus, the sustainable management of PPEs and other medical waste from COVID-19 has become a key environmental challenge [41,95,98,108]. The lack of a harmonized international strategy and approach to manage PPE production and disposal as well as COVID-19 medical waste lifecycle threatens the advancement toward achieving key components of the United Nation’s Sustainable Development Goals (SDGs). These environmental challenges particularly impact on SDG -3 good health and wellbeing, SDG -6 (clean water and sanitation), SDG-8) decent work and economic growth), SDG-12 (responsible consumption and production), and SDG-13 (climate action) [17,95,98].

## 8. Results and Discussion

This study adopts a qualitative approach for studying the various management and disposal practices adopted in Australia for handling medical waste from the COVID-19 pandemic, and its impacts on public health and the environment. A secondary data from 2019–2020 has been considered for reviewing existing literature on medical waste from COVID-19. Finally, problems and challenges associated with managing COVID-19 waste are discussed and recommendations in the short and long term towards managing COVID-19 waste presented. 

### Analysis of Content Results

Given the background review and analysis in the previous sections, it is obvious that Australia adopts a multilevel system in the management of waste including medical waste from COVID-19 pandemic. Findings from the analysis show that the management of COVID-19 waste is primarily the responsibility of states and territories governments, which regulate and manage waste in accordance with their individual policies, legislation, and programs. The Commonwealth in collaboration with the Waste Management and Resource Recovery Association of Australia (WMRR) is responsible for national legislation, strategies, and policy frameworks that reflect obligations under international agreements [67,109]. Strategies to establish consistency in medical waste management through collaboration have existed since 1992 when the Commonwealth responded to problems of inconsistent approaches to waste management (including medical wastes) policy by state and local governments [67,110].

This study found that to ensure that COVID-19 waste transported within Australian states and territories are properly identified, treated, and disposed of in an ecologically sound manner, the National Environmental Protection Measure (NEPM) was formulated to supervise movement of controlled waste [67]. However, the extent to which the NEPM is applied is still decided by each jurisdiction. Moreover, in the states and territories, when new disposal service contracts are established, medical waste generators are often not educated of the different technologies used by the waste management agencies that may require different segregation practices. Notwithstanding the issues highlighted above, definitions and guidelines on medical waste management and disposal have been developed by each state and territory [15,67]. 

In addition, a manifest system for tracking the movement of COVID-19 waste is in place, and states and territories apply the polluter-pays principle (PPP), whereby healthcare facilities pay for COVID-19 waste management. Currently, strategies for effectively managing medical waste are being developed via the National Waste Policy in association with the Commonwealth, the National Environment Protection Council (NEPC), and individual states and territories governments. It is anticipated that management of medical waste from COVID-19 will improve as these guidelines are implemented [15,67]. 

The results of this analysis also show that due to the infectious nature of COVID-19 waste, all states and territories have to strictly adhere to occupational health and safety (OHS) requirements and practices based on the local, WHO, UNEP, and Basel Convention models. Medical staff and waste management staff are most at risk during COVID-19 pandemic since they are exposed to infection and injury from hazards, especially sharps. To protect medical workers, each state and territories’ government has to provide required PPEs for medical staff and training on personal protection, and inform and train medical staff on good hygiene practices and safety measures [61,66,67].

Although Australia has a well-developed and efficient healthcare system, with the rapidly rising number of confirmed cases in some states and territories and the amounts of COVID-19 waste generated in healthcare facilities and testing sites, achieving a sustainable and efficient management of COVID-19 waste has remained a challenge during the pandemic [7,15]. As a result, waste management agencies are currently optimizing their existing systems to accommodate the radical increase in COVID-19 waste. With the current lockdowns and restrictions in major states/territories and some regional areas, large amounts of household waste and infectious waste from home quarantine have also increased. This unexpected surge has put extraordinary pressure on existing medical waste management systems and requires drastic action by policy makers [15,55]. 

As previously noted, some categories of medical waste, including those from COVID-19 patients, are classified as infectious waste and are highly transmissible [27,52]. Due to the nature and transmissibility of infectious waste (including COVID-19 waste), nations across the world have clearly defined and differentiated between infectious medical waste and municipal waste. This differentiation has prompted separate methods and procedures for handling, treatment, and disposal [52,54]. Due to the increasing number of COVID-19 waste generated worldwide from healthcare establishments such as hospitals and laboratories, and other sources such as aged care facilities, home quarantine, temporary establishments, sample collection and testing centres, graveyards, and crematoriums [17,54], COVID-19 waste could comprise of both municipal waste as well as infectious medical waste depending on the source of its generation. Generally, any waste generated from households in which a person infected with COVID-19 virus has been isolated and is undergoing treatment from home is categorized as COVID-19 waste [54,65]. In contrast, medical waste generated from any healthcare facility that is not related to any COVID-19 activity including diagnosis, research, or treatment does not fall under COVID-19 waste [17,54]. Hence, management of COVID-19 waste requires increased attention and caution to prevent adverse health and environmental consequences due to exposure of infectious pathogens and toxic substances [54,58].

The results of this analysis further shows that the environmental impacts resulting from the current pandemic include changes in traffic and mobility trends, air pollution, noise level and pollution, and waste generation. These changes were particularly obvious in Melbourne and Sydney during the first and second wave of the pandemic. Findings indicate huge demand for PPEs, particularly masks and facial shields, during the pandemic. This has created enormous waste from PPEs; thus, the sustainable management of PPEs and other medical waste has become a key environmental challenge [6,7,60]. Although COVID-19 has had a huge impact on overall public health in Australia, the lowering of emissions and environmental pollution from various methods of transportation and heavy industrial and manufacturing equipment brought about a reduction in pollutants such as nitrogen dioxide (NO_2_) in the atmosphere, which is often responsible for some human diseases (inflammation and respiratory disease, hypertension, coronary heart disease, etc.) and environmental degradation [6]. Furthermore, findings show that there have been improvements in air quality and better visibility in states and territories due to the COVID-19 lockdowns and these changes are linked to a better quality of life and health. 

## 9. Conclusions, Recommendations, and Future Research

### 9.1. Conclusions

This study has reviewed the current COVID-19 situation in Australia, the management and disposal of COVID-19 waste, and the direct and indirect impacts of the pandemic on public health and the environment. Based on the literature review and analysis, the following medical waste management needs have been identified: (a) improve consistency in medical waste definitions in various jurisdictions; (b) develop national medical waste management procedures, guidelines, and regulations; (c) synchronise and streamline established technologies for the treatment of medical waste at the national level; (d) create a harmonized system for the supervision and monitoring of healthcare sector; and (e) improvement and innovation in the healthcare sector in response to future pandemics.

Thus, the COVID-19 pandemic has had a far-reaching impact on the world (including Australia) and has created a huge burden on healthcare systems. Apart from the health-related effects of COVID-19, there are further impacts of the lockdowns on all aspects of human life and the economy. Australia has adopted different strategies in response to the management of the huge amount of infectious waste produced during the COVID-19 pandemic. In response to the pandemic, healthcare waste management strategies included several additional measures to ensure appropriate containment to avoid transmission and spread of the disease. Australia has also adopted the best possible management approaches based on the nation’s capacity, resources, and commitment and is rated among countries with excellent containment of COVID-19. 

While lockdowns effectively reduced the transmission and spread of COVID-19, stringent control measures aimed at reducing disease mortality and morbidity are often accompanied by negative consequences in many sectors of the economy. The impacts are often multidimensional and long-term and, therefore, an important factor for policy makers to consider when choosing which intervention packages to implement in future disease outbreaks.

With the current situation in Australia and the challenges involved in managing medical waste from COVID-19 pandemic, this study provides insights on short and long term responses towards managing COVID-19 waste. The study contributes to Australia’s efforts against the transmission and spread of COVID-19 and provides alternative management approaches and recommendations for the development of workable and sustainable strategies for mitigating similar pandemics and disease outbreaks in the future. 

Finally, the current coronavirus pandemic has put the resilience of the government and people of Australia to the test. The COVID-19 pandemic has continued to exert extraordinary pressures on many economic activities, including those that are crucial to our well-being. Therefore, protecting human lives, livelihoods, and the environment are at the centre of all actions and decisions to deal with the pandemic at both individual and collective levels.

A limitation of this study is that it is limited to Australia. Although the accuracy of some of the analysis in the present study is inescapably subjective, this study is a starting point for further research into various aspects of medical waste management during the COVID-19 pandemic. 

### 9.2. Recommendations

Actions to ensure the safe and environmentally sound management of COVID-19 waste can prevent undesirable health and environmental impacts arising from the release of chemical or biological threats and drug-resistant pathogens into the environment, helping to protect the health of patients, medical workers, and the general public. To more-effectively manage COVID-19 waste, the Commonwealth government in partnership with the states and territories government should focus on short and long term priority areas that improve medical waste management to prevent the spread of COVID-19 and to develop resilience to and readiness for similar events in the future. The following recommendations are suggested based on the findings of this study.

#### 9.2.1. Short Term Responses

◦ All medical workers including formal municipality waste management employees should be encouraged to comply with OHS requirements and the mandatory use of PPEs.
◦ During disease outbreaks such as the COVID-19 pandemic, a robust arrangement is needed with clear roles and responsibilities assigned for the collection, treatment, and disposal of COVID-19 waste from healthcare facilities, households, and public places.
◦ During COVID-19 waste management, emergency response such as contingency plans should be developed as well as further improvement in medical waste management through review and enforcement of existing policies and regulatory frameworks.
◦ Ongoing assessment of the current medical waste systems to identify the capacities and gaps in the respective states and territories and increase the usage of recommended treatment technologies towards maximum capacity.
◦ Due to a surge in the generation of potentially infected PPEs (face masks, face shields, disposable plastics, etc.), further guidelines should be sought for COVID-19 waste generated from households, quarantine facilities, and other public places, in addition to international regulations and guidelines adopted for the safe management of COVID-19 waste.
◦ Proper segregation, packaging and storage of potentially infected materials using the approved double bag should be maintained. Collection frequency should be adjusted based on priority with possible reduction on collection of recyclables.
◦ Appropriate use of PPEs when handling COVID-19 waste, hand hygiene, as well as other precautionary practices to protect the health and safety of waste management workers.
◦ Since the informal sector plays a crucial role in managing COVID-19 waste, greater awareness on mitigating risks of coronavirus infection, social distancing, and handwashing should be continually emphasized.

#### 9.2.2. Long Term Responses

◦ Re-evaluation of the existing policies and regulations should be undertaken by the states’ and territories’ governments to appraise respective responses to COVID-19 waste management to better respond to similar future pandemics and clarify the actions that need to be taken.
◦ The current medical waste management system should be further organized with capabilities to handle household/quarantine COVID-19 waste with clear labelling and special bins.
◦ Innovative medical waste management infrastructure should be established with the required capacity and features to manage medical contaminated waste treatment to help overcome a similar disease outbreak or pandemic in the future.
◦ Financial resources and access to funding should be clearly identified to support the improvement of medical waste infrastructure.
◦ Public awareness and continuous training of medical staff and municipal employees on the positive impact of the appropriate management of hazardous or infectious medical waste should be enhanced.

### 9.3. Future Research

Future research should use a quantitative approach or other research methods to study medical waste from COVID-19 and its impact on public health and the environment. This will provide additional knowledge in understanding sustainable medical waste management during the pandemic and significantly facilitate the approach developed for dealing with similar possible pandemics in the future.

## Figures and Tables

**Table 1:** Categories of medical waste.

Medical Waste Categories	Description and Examples
Infectious waste	Waste suspected to contain pathogens, e.g., laboratory cultures; waste from isolation wards; tissues (swabs), materials, or equipment that have been in contact with infected patients; excreta
Pathological waste	Human tissues or fluids, e.g., body parts; blood and other bodily fluids; foetuses
Sharps	Sharp waste, e.g., needles, infusion sets, scalpels, knives, blades, broken glass
Pharmaceutical waste	Waste containing pharmaceuticals, e.g., pharmaceuticals that are expired or no longer needed, items contaminated by or containing pharmaceuticals (bottles, boxes)
Cytotoxic waste	Waste containing substances with cytotoxic properties, e.g., waste containing cytostatic drugs (often used in cancer therapy), cytotoxic chemicals
Chemical waste	Waste containing chemical substances, e.g., laboratory reagents, film developer, disinfectants that are expired or no longer needed, solvents
Wastes with high content of heavy metals	Batteries, broken thermometers, blood-pressure gauges, etc.
Pressurized containers	Gas cylinders, gas cartridges, aerosol cans
Radioactive waste	Waste containing radioactive substances, e.g., unused liquids from radiotherapy or laboratory research contaminated glassware, packages, or absorbent paper; urine and excreta from patients treated or tested with unsealed radionuclides; sealed sources

**Table 2:** Treatment and disposal methods for medical waste.

Waste Type	Incineration	Autoclaving and Shredding	Chemical Disinfection Using Hypochlorite and Shredding	Chemical Disinfection Using Peroxide, Lime and Shredding	Microwave and Shredding	Compaction	Landfilling
Infectious medical waste (untreated)	✔	✔	✔	✔	✔	✔ Other than animal carcass or sharps	✖ Other than in a scheduled area
Infectious medical waste (treated)	-	-	-	-	-	✔	✔
Sharps	-	-	✔	✔	-	-	-
Pathological waste	✔	✖	✖	✔	✖	✖	✖
Chemical waste	✔	✖	✖	✔	✖	✖	✖
Pharmaceutical waste	✔	✖	✖	✖	✖	✖	✖
Cytotoxic waste	✔	✖	✖	✖	✖	✖	✖
Radioactive waste	✖	✖	✖	✖	✖	✖	✖

Developed for this study. Data adapted from the WHO [71] and UNEP [66].

## Data Availability

Not applicable.

## References

[1] World Health Organisation (WHO) (2020). Coronavirus Disease (COVID-19) Pandemic. https://www.who.int/emergencies/diseases/novel-coronavirus-2019.

[2] United Nations Environment Programme (UNEP) (2020). Waste Management during COVID-19 Pandemic: From Response to Recovery. https://www.unenvironment.org/resources/report/waste-management-during-covid-19-pandemic-response-recovery.

[3] (2020). Centres for Disease Control and Prevention (CDCP) Considerations for Wearing Masks: Help Slow the Spread of COVID-19. https://www.cdc.gov/coronavirus/2019-ncov/prevent-getting-sick/cloth-face-cover-guidance.html.

[4] ADB (2020). Managing Infectious Medical Waste during the COVID-19 Pandemic. https://www.adb.org/publications/managing-medical-waste-covid19.

[5] Australian Government Department of Health (AGDH) (2021). Coronavirus Disease 2019 (COVID-19): CDNA National Guidelines for Public Health Units. https://www1.health.gov.au/internet/main/publishing.nsf/Content/cdna-song-novel-coronavirus.htm.

[6] Boroujeni M., Saberian M., Li J. (2021). Environmental impacts of COVID-19 on Victoria, Australia, witnessed two waves of Coronavirus. Environ. Sci. Pollut. Res..

[7] O’Sullivan D., Rahamathulla M., Pawar M. (2020). The impact and implications of COVID-19: An Australian perspective. Int. J. Community Soc. Dev..

[8] Guardian Australia (2021). Coronavirus Australia Numbers: How Many New Cases are There? COVID-19 Map, Charts, Statistics and Graphs. https://www.theguardian.com/australia-news/datablog/nginteractive/2021/sep/09/covid-19-australia-tracer-map-data-cases-today-coronavirus-tracking-stats-live-data-update-by-state-melbourne-regional-victoria-vic-sydney-nsw-how-many-new-active-case-numbers-statistics-deaths-death-toll.

[9] Guardian Australia (2020). Coronavirus Australia Numbers: How Many New Cases are There? COVID-19 Map, Charts, Statistics and Graphs. https://www.theguardian.com/australia-news/datablog/ng-interactive/2020/may/11/coronavirus-cases-australia-numbers-map-howmany-new-today-stats-statistics-charts-graphs-hotspots-by-postcode-covid-19-deathsdeath-toll.

[10] Jones S. (2020). Waste management in Australia is an environmental crisis: What needs to change so adaptive governance can help?. Sustainability.

[11] Ritchie M., Roser H. Our World in Data. Oxford Martin School, University of Oxford. https://ourworldindata.org/coronavirus-data.

[12] Sivey P. (2020). New Roadmap Gives Australia Two Paths Out Of COVID-19 Lockdown: Elimination or Adaptation. https://theconversation.com/new-roadmap-gives-australiatwo-paths-out-of-covid-19-lockdown-elimination-or-adaptation-137494.

[13] Codreanu T.A., Ngeh S., Trewin A., Armstrong P.K. (2021). Successful Control of an Onboard COVID-19 Outbreak Using the Cruise Ship as a Quarantine Facility, Western Australia, Australia. Emerg. Infect. Dis..

[14] Capoor M.R., Parida A. (2021). Biomedical Waste and Solid Waste Management in the Time of COVID-19: A Comprehensive Review of the National and International Scenario and Guidelines. J. Lab. Physicians.

[15] Scott N., Saul A., Spelman T., Stoove M., Pedrana A., Saeri A., Grundy E., Smith L., Toole M., McIntyre C.R. (2021). The introduction of a mandatory mask policy was associated with significantly reduced COVID-19 cases in a major metropolitan city. PLoS ONE.

[16] Hu B., Guo H., Zhou P., Shi Z.L. (2021). Characteristics of SARS-CoV-2 and COVID-19. Nat. Rev. Microbiol..

[17] Singh N., Tang Y., Ogunseitan O.A. (2020). Environmentally sustainable management of used personal protective equipment. Environ. Sci. Technol..

[18] World Health Organisation (WHO) (2020). Rational Use of Personal Protective Equipment (PPE) For Coronavirus Disease (COVID-19). https://apps.who.int/iris/bitstream/handle/10665/331498/WHO-2019-nCoV-IPCPPE_use-2020.2-eng.pdf.

[19] United Nations Environment Programme (UNEP) (2020). Global Waste Management Outlook|UNEP—UN Environment Programme. https://www.unep.org/resources/report/global-waste-management-outlook.

[20] Saul A., Scott N., Crabb B.S., Majumdar S.S., Coghlan B., Hellard M.E. (2020). Impact of Victoria’s Stage 3 lockdown on COVID-19 case numbers. Med. J. Aust..

[21] Harapan H., Itoh N., Yufika A., Winardi W., Keam S., Te H., Mehwati D., Hayati Z., Wagner A.L., Mudatsir M. (2020). Coronavirus disease 2019 (COVID-19): A literature review. J. Infect. Public Health.

[22] United Nations Environment Programme (UNEP) (2020). COVID-19 Waste Management Factsheets. http://wedocs.unep.org/bitstream/handle/20.500.11822/32779/FSSum.pdf?sequence=1&isAllowed=y.

[23] Nzediegwu C., Chang S.X. (2020). Improper solid waste management increases potential for COVID-19 spread in developing countries. Resour. Conserv. Recycl..

[24] Mihai F.C. (2020). Assessment of COVID-19 waste flows during the emergency state in Romania and related public health and environmental concerns. Int. J. Environ. Res. Public Health.

[25] United Nations Environment Programme (UNEP) (2020). Waste Management an Essential Public Service in the Fight to Beat COVID-19. https://www.unep.org/news-and-stories/press-release/waste-management-essential-public-service-fight-beat-covid-19.

[26] Saadat S., Rawtani D., Hussain C.M. (2020). Environmental perspective of COVID-19. Sci. Total Environ..

[27] Hu L., Deng W.J., Ying G.G., Hong H. (2021). Environmental perspective of COVID-19: Atmospheric and wastewater environment in relation to pandemic. Ecotoxicol. Environ. Saf..

[28] Corburn J., Vlahov D., Mberu B., Riley L., Caiaffa W.T., Rashid S.F., Ko A., Patel S., Jukur S., Martinez-Herera E. (2020). Slum health: Arresting COVID-19 and improving well-being in urban informal settlements. J. Urban Health.

[29] Hasan M.M., Rahman M.H. (2018). Assessment of healthcare waste management paradigms and its suitable treatment alternative: A case study. J. Environ. Public Health.

[30] Shareefdeen Z.M. (2012). Medical waste management and control. J. Environ. Prot..

[31] Khan B.A., Cheng L., Khan A.A., Ahmed H. (2019). Healthcare waste management in Asian developing countries: A mini review. Waste Manag. Res..

[32] Malekahmadi F., Yunesian M. (2014). Analysis of the healthcare waste management status in Tehran hospitals. J. Environ. Health Sci. Eng..

[33] Nwachukwu N.C., Orji F.A., Ugbogu O.C. (2013). Health care waste management–public health benefits, and the need for effective environmental regulatory surveillance in Federal Republic of Nigeria. Curr. Top. Public Health.

[34] Hossain M.S., Santhanam A., Norulaini N.A. (2011). Clinical solid waste management practices and its impact on human health and environment—A review. Waste Manag..

[35] Patwary M.A., O’Hare W.T., Sarker M.H. (2011). Assessment of occupational and environmental safety associated with medical waste disposal in developing countries: A qualitative approach. Saf. Sci..

[36] Gupta S., Boojh R. (2006). Report: Biomedical waste management practices at Balrampur Hospital, Lucknow, India. Waste Manag. Res..

[37] Wang D., Hu B., Hu C., Zhu F., Liu X., Zhang J., Wang B., Xiang H., Cheng Z., Xiong Y. (2020). Clinical characteristics of 138 hospitalized patients with 2019 novel coronavirus–infected pneumonia in Wuhan, China. JAMA.

[38] Akter N., Trankler J. (2003). An analysis of possible scenarios of medical waste management in Bangladesh. Manag. Environ. Qual. Int. J..

[39] Shinee E., Gombojav E., Nishimura A., Hamajima N., Ito K. (2008). Healthcare waste management in the capital city of Mongolia. Waste Manag..

[40] Wilder-Smith A., Freedman D.O. (2020). Isolation, quarantine, social distancing and community containment: Pivotal role for old-style public health measures in the novel coronavirus (2019-nCoV) outbreak. J. Travel Med..

[41] United States Environmental Protection Agency (USEPA) (2008). EPA’S Report on the Environment (ROE).

[42] Peng J., Wu X., Wang R., Li C., Zhang Q., Wei D. (2020). Medical waste management practice during the 2019–2020 novel coronavirus pandemic: Experience in a general hospital. Am. J. Infect. Control..

[43] Sutha Irin A. (2018). An analytical study on medical waste management in selected hospitals located in Chennai city. Environ. Waste Manag. Recycl..

[44] EPA South Australia (2021). Medical Waste Definition. https://www.epa.sa.gov.au/community/waste_and_recycling/medical_waste.

[45] World Health Organisation (WHO) (2020). Infection Prevention and Control for the Safe Management of a Dead Body in the Context of COVID-19: Interim Guidance.

[46] United States Environmental Protection Agency (USEPA) (2021). Medical Waste. https://www.epa.gov/rcra/medical-waste.

[47] Abd El-Salam M.M. (2010). Hospital waste management in El-Beheira Governorate, Egypt. J. Environ. Manag..

[48] Ananth A., Prashanthini V., Visvanathan C. (2010). Healthcare waste management in Asia. Waste Manag..

[49] World Health Organization (WHO) (2005). Management of Solid Health-Care Waste at Primary Health-Care Centres. http://www.who.int/water_sanitation_health/publications/manhcwm.pdf.

[50] World Health Organisation (WHO) (2020). Rational Use of Personal Protective Equipment For Coronavirus Disease (COVID-19) and Considerations During Severe Shortages. https://apps.who.int/iris/bitstream/handle/10665/331695/WHO-2019-nCov-IPC_PPE_use-2020.3-eng.pdf.

[51] World Health Organisation (WHO) (2017). Safe Management of Wastes from Health-Care Activities: A Summary.

[52] Mol M.P.G., Caldas S. (2020). Can the human coronavirus epidemic also spread through solid waste?. Waste Manag. Res..

[53] Chen Y., Guo C. (2020). Handbook of Emergency Disposal and Management of Medical Waste in China.

[54] Sharma H.B., Vanapalli K.R., Cheela V.R.S., Ranjan V.P., Jaglan A.K., Dubey B., Goel S., Bhattacharya J. (2020). Challenges, opportunities, and innovations for effective solid waste management during and post COVID-19 pandemic. Resour. Conserv. Recycl..

[55] Abu-Qdais H.A., Al-Ghazo M.A., Al-Ghazo E.M. (2020). Statistical analysis and characteristics of hospital medical waste under novel Coronavirus outbreak. Glob. J. Environ. Sci. Manag..

[56] Graff K., Smith C., Silveira L., Jung S., Curran-Hays S., Jarjour J., Carpenter L., Pickard K., Mattiucci M., Fresia J. (2021). Risk factors for severe COVID-19 in children. Pediatr. Infect. Dis. J..

[57] Qiao J. (2020). What are the risks of COVID-19 infection in pregnant women?. Lancet.

[58] Scheinberg A., Woolridge A., Humez N., Mavropoulos A., Filho C.S., Savino A., Ramola A. (2020). Waste Management During the COVID-19 Pandemic.

[59] Chen K., Wang M., Huang C., Kinney P.L., Anastas P.T. (2020). Air pollution reduction and mortality benefit during the COVID-19 outbreak in China. Lancet Planet. Health.

[60] Salvaraji L., Jeffree M.S., Avoi R., Atil A., Akhir H.M., Shamsudin S.B.B., Lukman K.A. (2020). Exposure risk assessment of the municipal waste collection activities during COVID-19 pandemic. J. Public Health Res..

[61] World Health Organisation (WHO) (2020). WHO Announces COVID-19 Outbreak a Pandemic. https://www.euro.who.int/en/health-topics/health-emergencies/coronavirus-covid-19/news/news/2020/3/whoannounces-covid-19-outbreak-a-pandemic.

[62] Koh D. (2020). Occupational risks for COVID-19 infection. Occup. Med..

[63] Sarkodie S.A., Owusu P.A. (2020). Impact of meteorological factors on COVID-19 pandemic: Evidence from top 20 countries with confirmed cases. Environ. Res..

[64] International Finance Corporation (IFC) (2020). COVID-19’s Impact on the Waste Sector. https://www.ifc.org/wps/wcm/connect/industry_ext_content/ifc_external_corporate_site/infrastructure/resources/covid-19-and-waste-sector.

[65] Chen N., Zhou M., Dong X., Qu J., Gong F., Han Y., Qiu Y., Wang J., Liu Y., Wei Y. (2020). Epidemiological and clinical characteristics of 99 cases of 2019 novel coronavirus pneumonia in Wuhan, China: A descriptive study. Lancet.

[66] United Nations Environment Programme (UNEP) (2020). Healthcare or Medical Waste: Basel Convention on the Control of Transboundary Movements of Hazardous Wastes and Their Disposal. http://www.basel.int/?tabid=5839.

[67] Department of Environment and Energy (DEE) (2020). Policies and Governance of Waste. https://www.environment.gov.au/protection/waste-resource-recovery/national-wastereports/national-waste-report-2013/policies-and-governance.

[68] World Health Organisation (WHO) (2015). Status of Health-Care Waste Management in Selected Countries of The Western Pacific Region.

[69] Western Australia Department of Health (2021). Code of Practice for Clinical and Related Waste Management Public Health Act 2016. https://ww2.health.wa.gov.au/Articles/A_E/Code-of-practice-for-clinical-and-related-waste-management.

[70] Standards Australia Management of Clinical and Related Wastes, 2018, AS 3816:2018. https://www.standards.org.au/standards-catalogue/sa-snz/health/he-011/as--3816-colon-2018.

[71] World Health Organisation (WHO) (2020). Water, Sanitation, Hygiene, and Waste Management for the COVID-19 Virus: Interim Guidance.

[72] European Commission (2020). Waste Management in The Context of the Coronavirus Crisis.

[73] United States Occupational Health and Safety (OSHA) (2020). COVID-19—Control and Prevention—Solid Waste and Wastewater Management Workers and Employers|Occupational Safety and Health Administration. https://www.osha.gov/coronavirus/control-prevention/solid-waste-wastewater-mgmt.

[74] CDC (2019). Guidelines for Environmental Infection Control in Health-Care Facilities Recommendations of CDC and the Healthcare Infection Control Practices Advisory Committee (HICPAC).

[75] CPCB (2020). Revision 4 Guidelines for Handling, Treatment and Disposal of Waste Generated during Treatment/Diagnosis/Quarantine of COVID-19.

[76] Australian Government Department of Health (AGDH) (2020). Coronavirus Disease (COVID-19): Environmental Cleaning And Disinfection Principles for COVID-19. https://www.health.gov.au/sites/default/files/documents/2020/03/environm.

[77] Das A.K., Islam N., Billah M., Sarker A. (2021). COVID-19 pandemic and healthcare solid waste management strategy—A mini-review. Sci. Total Environ..

[78] Al-Khatib I.A., Eleyan D., Garfield J. (2016). A system dynamics approach for hospital waste management in a city in a developing country: The case of Nablus, Palestine. Environ. Monit. Assess..

[79] Ciplak N., Barton J.R. (2012). A system dynamics approach for healthcare waste management: A case study in Istanbul Metropolitan City, Turkey. Waste Manag. Res..

[80] Government of South Australia (SA Health) (2021). COVID-19 Health Information. https://www.sahealth.sa.gov.au/wps/wcm/connect/public+content/sa+health+internet/conditions/infectious+diseases/covid-19/covid-19.

[81] Victorian Department of Health and Human Services (VDHHS) (2021). Victorian Coronavirus (COVID-19) Data. https://www.dhhs.vic.gov.au/victoriancoronavirus-covid-19-data.

[82] Rao S., Ranyal R.K., Bhatia S.S. (2004). Biomedical waste management: An infrastructural survey of hospitals. Med. J. Armed Forces India.

[83] Patil A.D., Shekdar A.V. (2001). Health-care waste management in India. J. Environ. Manag..

[84] Australian Government Department of Health (AGDH) (2021). Coronavirus (COVID-19) at a Glance Infographic Collection. https://www.health.gov.au/resources/collections/coronavirus-covid-19-at-aglance-infographic-collection.

[85] Queensland Department of Environment and Science (2019). Clinical and Related Wastes.

[86] Onyeaka H., Anumudu C.K., Al-Sharify Z.T., Egele-Godswill E., Mbaegbu P. (2021). COVID-19 pandemic: A review of the global lockdown and its far-reaching effects. Sci. Prog..

[87] Cheng V.C.C., Wong S.C., Chuang V.W.M., So S.Y.C., Chen J.H.K., Sridhar S., To K.K.W., Chan J.F.W., Hung I.F., Ho P.L. (2020). The role of community-wide wearing of face mask for control of coronavirus disease 2019 (COVID-19) epidemic due to SARS-CoV-2. J. Infect..

[88] Ma Y., Lin X., Wu A., Huang Q., Li X., Yan J. (2020). Suggested guidelines for emergency treatment of medical waste during COVID-19: Chinese experience. Waste Dispos. Sustain. Energy.

[89] Sangkham S. (2020). Face mask and medical waste disposal during the novel COVID-19 pandemic in Asia. Case Stud. Chem. Environ. Eng..

[90] He G., Pan Y., Tanaka T. (2020). The short-term impacts of COVID-19 lockdown on urban air pollution in China. Nat. Sustain..

[91] Dutheil F., Baker J.S., Navel V. (2020). COVID-19 as a factor influencing air pollution?. Environ. Pollut..

[92] Ameer W., Xu H. (2020). COVID-19 Pandemic and Environmental Pollution. Int. J. Environ. Sci. Nat. Resour..

[93] Liu Z., Ciais P., Deng Z., Lei R., Davis S.J., Feng S., Zheng B., Cui B., Duo X., Zhu B. (2020). Near-real-time monitoring of global CO_2_ emissions reveals the effects of the COVID-19 pandemic. Nat. Commun..

[94] Muhammad S., Long X., Salman M. (2020). COVID-19 pandemic and environmental pollution: A blessing in disguise?. Sci. Total Environ..

[95] Almond R.E.A., Grooten M., Petersen T., World Wildlife Fund (WWF) (2020). Living Planet Report 2020—Bending the Curve of Biodiversity Loss.

[96] Rowan N.J., Laffey J.G. (2020). Unlocking the surge in demand for personal and protective equipment (PPE) and improvised face coverings arising from coronavirus disease (COVID-19) pandemic—Implications for efficacy, re-use and sustainable waste management. Sci. Total Environ..

[97] Zambrano-Monserrate M.A., Ruano M.A., Sanchez-Alcalde L. (2020). Indirect effects of COVID-19 on the environment. Sci. Total Environ..

[98] UNCTAD (2020). COVID-19 Stalls Progress on Global Goals. https://unctad.org/news/covid-19-stalls-progress-global-goals.

[99] Jiang P., Fu X., Fan Y.V., Klemes J.J., Chen P., Ma S., Zhang W. (2020). Spatial-temporal potential exposure risk analytics and urban sustainability impacts related to COVID-19 mitigation: A perspective from car mobility behaviour. J. Clean. Prod..

[100] Giani P., Castruccio S., Anav A., Howard D., Hu W., Crippa P. (2020). Short-term and long-term health impacts of air pollution reductions from COVID-19 lockdowns in China and Europe: A modelling study. Lancet Planet. Health.

[101] Gautam S. (2020). COVID-19: Air pollution remains low as people stay at home. Air Qual. Atmos. Health.

[102] Prata J.C., Silva A.L., Walker T.R., Duarte A.C., Rocha-Santos T. (2020). COVID-19 pandemic repercussions on the use and management of plastics. Environ. Sci. Technol..

[103] Benson N.U., Bassey D.E., Palanisami T. (2020). COVID pollution: Impact of COVID-19 pandemic on global plastic waste footprint. Heliyon.

[104] Herron J.B.T., Hay-David A.G.C., Gilliam A.D., Brennan P.A. (2020). Personal protective equipment and Covid-19-a risk to healthcare staff?. Br. J. Oral Maxillofac. Surg..

[105] European Centre for Disease Prevention and Control (ECDC) (2020). Using Face Masks in the Community Stockholm. https://www.ecdc.europa.eu/en/publications-data/using-face-masks-community-reducing-covid-19-transmission.

[106] Tang A.N., Tong Z.D., Wang H.L., Dai Y.X., Li K.F., Liu J.N., Wu W.J., Yuan C., Yu M., Li P. (2020). Detection of novel coronavirus by RT-PCR in stool specimen from asymptomatic child, China. Emerg. Infect. Dis..

[107] Lodder W., de Roda Husman A.M. (2020). SARS-CoV-2 in wastewater: Potential health risk, but also data source. Lancet Gastroenterol. Hepatol..

[108] Fadare O.O., Okoffo E.D. (2020). Covid-19 face masks: A potential source of micro plastic fibers in the environment. Sci. Total Environ..

[109] Andeobu L.N., Wibowo S., Grandhi S. (2021). An assessment of e-waste generation and environmental management of selected countries in Africa, Europe and North America: A systematic review. Sci. Total Environ..

[110] Andeobu L.N., Wibowo S., Grandhi S. (2021). A systematic review of e-waste generation and environmental management of Asia Pacific countries. Int. J. Environ. Res. Public Health.

